# Epidemiology of musculoskeletal injury in military recruits: a systematic review and meta-analysis

**DOI:** 10.1186/s13102-023-00755-8

**Published:** 2023-10-28

**Authors:** Myles C. Murphy, Joanne Stannard, Vanessa R. Sutton, Patrick J. Owen, Brendon Park, Paola T. Chivers, Nicolas H. Hart

**Affiliations:** 1 Nutrition and Health Innovation Research Institute, School of Medical and Health Sciences, Edith Cowan University, Joondalup, WA Australia; 2 School of Health Sciences and Physiotherapy, The University of Notre Dame Australia, Fremantle, WA Australia; 3 School of Medical and Health Sciences, Edith Cowan University, Joondalup, WA Australia; 4 Institute for Physical Activity and Nutrition (IPAN), School of Exercise and Nutrition Sciences, Deakin University, Geelong, VIC Australia; 5 Institute for Health Research, The University of Notre Dame Australia, Fremantle, WA Australia; 6 Caring Futures Institute, College of Nursing and Health Science, Flinders University, Adelaide, SA Australia; 7 School of Nursing, Faculty of Health, Queensland University of Technology, Brisbane, QLD Australia

**Keywords:** Injury epidemiology, Navy, Marine, Army, Air force, Injury surveillance

## Abstract

**Background:**

Injuries are a common occurrence in military recruit training, however due to differences in the capture of training exposure, injury incidence rates are rarely reported. Our aim was to determine the musculoskeletal injury epidemiology of military recruits, including a standardised injury incidence rate.

**Methods:**

Epidemiological systematic review following the PRISMA 2020 guidelines. Five online databases were searched from database inception to 5^th^ May 2021. Prospective and retrospective studies that reported data on musculoskeletal injuries sustained by military recruits after the year 2000 were included. We reported on the frequency, prevalence and injury incidence rate. Incidence rate per 1000 training days (Exact 95% CI) was calculated using meta-analysis to allow comparisons between studies. Observed heterogeneity (e.g., training duration) precluded pooling of results across countries. The Joanna Briggs Institute Quality Assessment Checklist for Prevalence Studies assessed study quality.

**Results:**

This review identified 41 studies comprising 451,782 recruits. Most studies (*n* = 26; 63%) reported the number of injured recruits, and the majority of studies (*n* = 27; 66%) reported the number of injuries to recruits. The prevalence of recruits with medical attention injuries or time-loss injuries was 22.8% and 31.4%, respectively. Meta-analysis revealed the injury incidence rate for recruits with a medical attention injury may be as high as 19.52 injuries per 1000 training days; and time-loss injury may be as high as 3.97 injuries per 1000 training days. Longer recruit training programs were associated with a reduced injury incidence rate (*p* = 0.003). The overall certainty of the evidence was low per a modified GRADE approach.

**Conclusion:**

This systematic review with meta-analysis highlights a high musculoskeletal injury prevalence and injury incidence rate within military recruits undergoing basic training with minimal improvement observed over the past 20 years. Longer training program, which may decrease the degree of overload experienced by recruit, may reduce injury incidence rates. Unfortunately, reporting standards and reporting consistency remain a barrier to generalisability.

**Trial registration:**

PROSPERO (Registration number: CRD42021251080).

**Supplementary Information:**

The online version contains supplementary material available at 10.1186/s13102-023-00755-8.

## Introduction

Military recruits, like other tactical operators [1], undergo strenuous physical conditioning during basic training to become qualified military personnel [2]. Initial military qualification courses often differ between countries based on duration [3–7] and whether service is voluntary or mandatory [6, 8]. Training programs can also vary by including only basic training [9] or combine basic training and trade training [10]. Further differences include the level of conditioning required by recruits, which often varies by service (e.g., air force, army, navy or marine), [11–13] and whether they are full- or part-time recruits [14]. Regardless of these differences in qualification training, military recruit training programs in all countries are burdened by musculoskeletal injuries [5, 9, 13, 15–20].

Injuries in military recruits account for a substantial amount of time-loss from basic training [4] and can result in medical discharge or delays in completion of qualification training [21]. Reduced graduate numbers impact qualified soldier availability and collectively impacts military capability. Furthermore, injuries impose substantial financial burden on military organisations and compensation systems. Prior research has indicated that over a seven-year period, recruit injuries during basic training within the United States airforce cost over $43.7 million USD. The scale and magnitude of the financial and health burden is compounded when considering other countries (i.e., beyond the United States) and military recruits from other professions (e.g., army recruits) [22]. For these reasons, injury mitigation is repeatedly highlighted as an organisational and research priority to protect personnel health and preserve military capability [23].

Currently, there is no international consensus guiding the recording and reporting of musculoskeletal injury epidemiology in military recruits [24]. Therefore, navigating the literature in this space can be confusing and challenging to translate the research findings into clinical practice. However, guidance can be obtained from an international consensus statement of recommendations for reporting of epidemiological data in physical activity and sport published in 2020 [25], with more specific reporting guidelines in military populations released in 2022 [26]. These guidelines are the product of increasing attention being afforded to the improvement of injury surveillance methods in military populations in order to encourage a consistent and comprehensive approach to collecting injury data to direct mitigation efforts and improve knowledge-sharing between nations [26]. These publications guided our definition of injury, the data we opted to extract, and how we reported results.

The purpose of this systematic review with meta-analysis is to adapt recent injury surveillance guidelines in the military to quantify the frequency, prevalence and incidence of musculoskeletal injuries sustained by military recruits to guide translation into policy and practice. Furthermore, we aimed to calculate the injury incidence based on estimated training duration, to provide the first review allowing between country, and service comparisons.

### Objectives

Determine the musculoskeletal injury epidemiology among military recruits.

## Methods

### Guidelines

This systematic review was designed and reported in accordance with the Preferred Reporting Items for Systematic Review and Meta-analyses (PRISMA) [27], and recent military consensus guidelines on musculoskeletal injury surveillance [26].

### Prospective registration

Prospectively registered with PROSPERO (CRD42021251080) [https://www.crd.york.ac.uk/prospero/].

### Data management

Covidence (Veritas Health Innovation, Melbourne, Australia) was used to record and store data related to study selection. Extracted data were inputted into Microsoft Excel and stored using Microsoft Teams and password-protected laptop computers.

### Criteria for considering studies for this review

#### Types of studies

Prospective and retrospective studies were included. This included cross-sectional and longitudinal studies and randomised controlled trials of injury prevention interventions. For example, randomised controlled trials that examined injury prevention (e.g., the effect of an injury prevention program in preventing injuries within military recruits) were included, provided they had a control arm without an intervention (i.e., the control arm was included within this review).

Only published studies were included within this review (i.e., grey literature was excluded). Non-English language studies were also excluded. Prior work suggested that inclusion or exclusion of non-English articles do not influence the effect estimates, yet may narrow confidence intervals [28].

#### Types of recruits

We included military recruits of any military service (i.e., air force, army, navy, and marine), entrance type (mandatory or voluntary, reserves or full-time), sex, and geographical location. Studies that solely included recruits from before the year 2000 were excluded as current training procedures and policies are likely to be substantially different from those implemented more than 20-years ago [2, 29, 30].

#### Types of injuries

Studies that reported data inclusive of musculoskeletal injury of all regions and injury types in military recruits were included. Studies that only recorded specific injury types (e.g., the study only recorded bone stress fracture or ankle ligament sprains) were excluded as they would bias injury frequency, prevalence, and incidence. Specifically, whilst recognising that studies assessing one single injury type have their place, as recruits who sustained injuries to other regions who be incorrectly classified as ‘un-injured’ for the purposes of this review and could not be pooled. All injury case definitions were included, such as all injury, medical attention injury, time-loss injury, or injury resulting in a medical discharge.

### Search methods for identification of studies

A single study author (MCM) implemented search strategies from inception until the 5^th^ of May 2021 and exported the records into Covidence.

#### Electronic searches

Searches were performed using free text and MeSH terms (Appendix A) within the following electronic databases: PubMed, CINAHL, CENTRAL, SPORTDiscus, and Web of Science. Peer review, English language and human trials were included as limiters; however, they were modified for each database as necessary (Appendix B). The search strategy was informed by a prior systematic review [31].

#### Searching other resources

Backwards citation tracking was performed via Web of Science and a screening of relevant reviews [32, 33] to identify studies missed by the search strategy. Studies available online, yet not indexed were also screened via the available online first section of key journal websites.

#### Selection of Studies

Pairs of two contributors (VRS/BP, VRS/MCM, or VRS/MM) independently assessed the titles and abstracts of potential studies identified by the search strategy for their eligibility. Studies also proceeded to full-text screening when the eligibility of a study was unclear from the title and abstract. Pairs of two contributors (VRS/BP or VRS/MCM) also independently assessed the full-text record of potential studies identified by the search strategy for their eligibility. Full-text studies which did not meet the inclusion criteria were excluded, and the reasons for exclusion were documented [34]. Disagreements between authors regarding study inclusion were resolved by discussion. Studies were not anonymised prior to assessment. This process was performed within Covidence.

### Data management 

#### Data extraction

Pairs of two study authors (VRS/MCM or BP/MCM) extracted data from included studies independently using a Microsoft Excel spreadsheet. Disagreements were resolved by consensus between review authors. The following items were extracted from full-text records: primary author, year of publication, country of origin, funding source, study design (retrospective or prospective data collection), military service (air force, army, navy or marines), sample size (n), duration of recruit training (weeks), mean (SD) baseline demographics for all recruits and injured recruits (age (years), sex (male/female), height (cm), weight (kg) and body mass index (BMI) (kg/m^2^), as well as the injury case definition (all injury, medical-attention injury, time-loss injury or injury requiring medical discharge), the number of injured recruits and the number of injuries.

#### Dealing with missing data

Where a method of exposure was not provided, the study was excluded from the injury incidence rate analysis. Authors were not contacted to request missing data as studies reported data from injury databases, indicating further data was unlikely to be available.

#### Dealing with multiple records representing a single trial

Where multiple identified studies used the same dataset, these were pooled to represent a single record, with the first study published being assigned as the reference study. Sharma et al. 2015 and 2017 used the same dataset; therefore, Sharma et al. 2015 was used as the primary study reported within this systematic review [10, 35]. Sharma et al. 2011 and 2019 used the same dataset; thus, Sharma et al. 2011 was used as the primary study [7, 36]. Cowan et al. 2011 and Bedno et al. 2013 used the same dataset, so Cowan et al. 2011 was used as the primary study [15, 37].

### Assessment of quality in included studies

The quality of included studies was independently assessed by two review authors (MCM and JS) using the Joanna Briggs Institute (JBI) Quality Assessment Checklist for Prevalence Studies. The appraisal items and criteria used to assess these items is presented in Appendix C. Studies were assessed against these checklist items as ‘yes’, ‘no’, or ‘unclear’. An overall high-quality rating was awarded if six or more checklist items were categorised as ‘yes’. Quality assessment results were compared between reviewers, and disagreements were resolved by discussion.

### Assessment of diversity and heterogeneity

Our protocol included a statistical assessment of heterogeneity between studies to explore the total variation across all included studies.

### Assessment of reporting biases 

The potential influence of small study biases, especially given that we allowed control arms from randomised controlled trials within our study was considered. Sample size bias in this review was in relation to the number of injuries, whereas the JBI checklist assessed the overall sample size. As previously reported, the influence of small study biases can be highlighted by the criterion ‘study size’ [31]. Specifically, studies with fewer than 50 injuries were considered as representing a high risk of study size bias, studies with between 50 and 200 injuries were classified as a moderate risk of study size bias and studies with greater than 200 injuries were classed as a low risk of study size bias [38].

### Assessment of the Certainty of the Body of Evidence 

Assessing the certainty of the body of evidence in systematic epidemiological reviews is different to systematic reviews of interventions, and the Grading of Recommendations Assessment, Development and Evaluation (GRADE) approach may be adjusted for different models (e.g., exposure) [39]. Within our review, we adapted the GRADE approach as per existing recommendations [39] for use in our epidemiological systematic review. Therefore, our judgement of the certainty of the body of evidence was based upon the number of injuries per study, overall study quality, indirectness, and inconsistency.

### Data synthesis 

Demographic data were described using count, mean (M), standard deviation (SD) or percentage (%), as appropriate. Injury prevalence was presented as a percentage, with the exposure denominator as the recruit training duration for the number of injuries. Evident clinical diversity of the population groups was seen between countries (e.g., duration of training or structure of training) precluded data pooling of all studies and subsequent statistical analysis of heterogeneity.

The injury incidence rate for the number of injuries was presented as the number of injuries per measure of exposure. Injury incidence rates were calculated per 1000 training days (Exact 95% CI) to allow meaningful comparisons between studies. Only studies that reported initial as well as subsequent/ recurrent injuries were included for analysis of injury incidence within this manuscript. However, we recognise many studies do not report these data and present the results for the injured recruit injury incidence rate within Appendix D.

Training days were selected as the measure for ‘exposure’. Exposure was calculated based on one week of recruit training representing six days of exposure, as this is the number of training days/week most commonly reported within individual studies. The pooled injury incidence rate per country was calculated, injuries/1000 training days (Exact 95% CI), as training regimes were comparable. As previously reported, clear clinical diversity of the populations used between studies precluded a meta-analysis (including sensitivity analysis) of all studies.

#### Subgroup analysis

At a study level, without pooling data, the association between the medical attention injury incidence rate (95% CI), and the duration of the training program (weeks) was assessed using a generalised linear model within SPSS Statistics Version 28.0.1.0 and all studies that reported the injury incidence rate were included (*n* = 26) and statistical significance was set at p < 0.05. Time-loss studies were not included due to the small number (*n* = 3). Model fit was assessed using the Akaike’s Information Criterion (AIC).

## Results

### Selection of studies

**Fig. 1:**
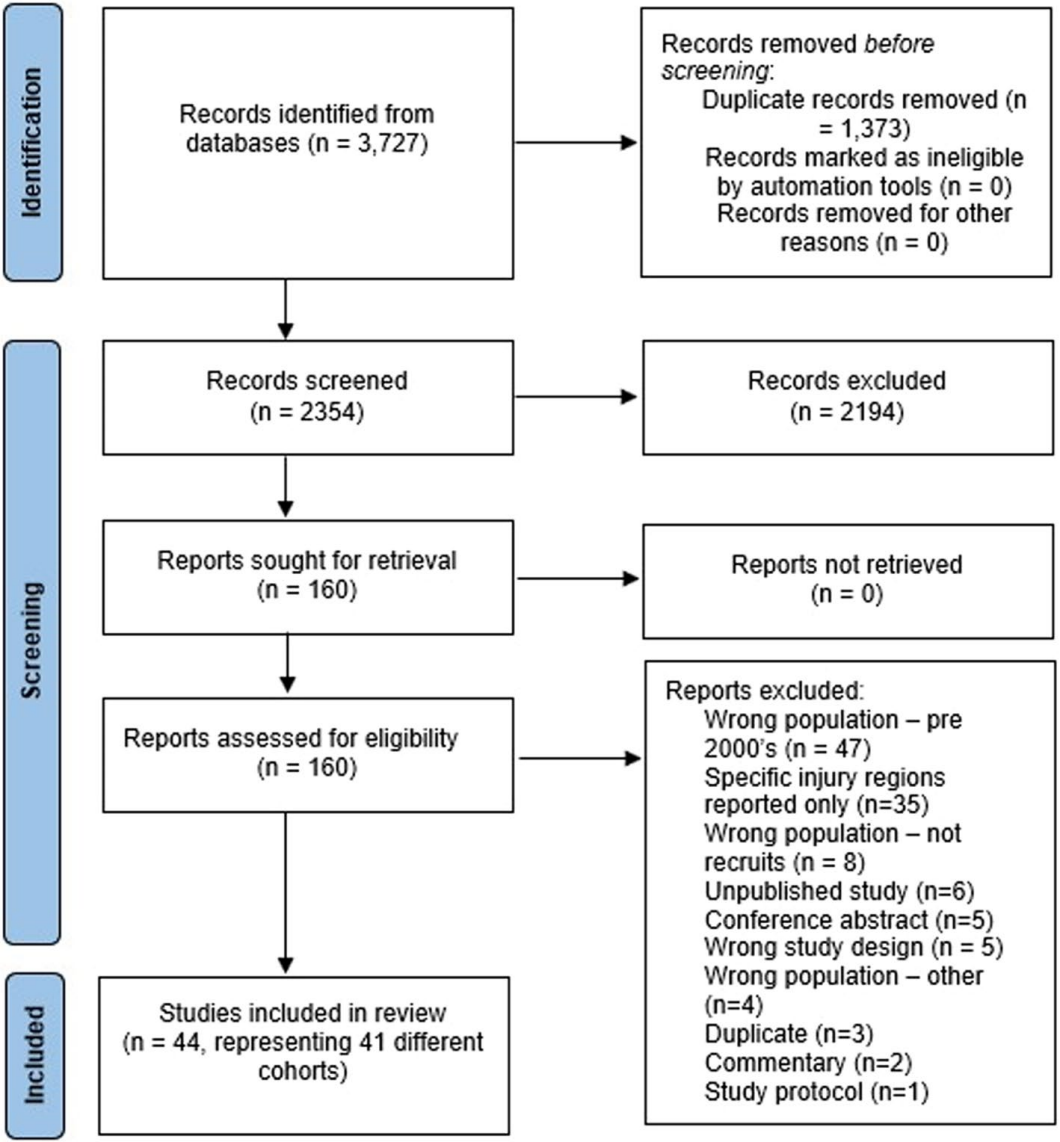
PRISMA flow chart

Collectively, 3,727 records were identified. After full-text screening, 44 records, representing 41 studies after combining publications that used shared datasets, met predefined eligibility criteria (Fig. 1) [3–20, 22, 35–37, 40–61].

### Study information 

Included studies reported injuries across military services: air force (*n* = 6;14.6%), army (*n* = 30;73.1%) and marines (*n* = 4;9.8%). One study did not clarify details regarding service [16]. An overview of study data is provided in Table 1. Geographical locations included the United States of America (*n* = 12;29.3%) [8, 19, 20, 22, 37, 40, 41, 44, 53–55, 58], the United Kingdom (*n* = 9;22.0%) [4, 7, 10–13, 18, 57, 59], Australia (*n* = 7;17.1%) [3, 9, 14, 17, 43, 45, 48], Switzerland (*n* = 3;7.3%) [6, 60, 61], Greece (*n* = 2;4.9%) [49, 50], Ireland (*n* = 2;4.9%) [47, 52], Canada (*n* = 1;2.4%) [16], Denmark (*n* = 1;2.4%) [42], Germany (*n* = 1;2.4%) [5], Iran (*n* = 1;2.4%) [56], Malaysia (*n* = 1;2.4%) [46], and Sweden (*n* = 1;2.4%) [51]. Recruit training periods ranged from 6–32 weeks [3, 4, 6–20, 22, 35–37, 40–43, 45–61], with two studies not reporting the training period duration [5, 44]. Data collection for most studies (*n* = 37;90%) was prospective (e.g., medical records), however, injury records were obtained retrospectively from the relevant military organisation. Funding sources are presented within Appendix E with few studies (*n* = 18/44;40.9%) reporting funding, which tended to be self-funded via the military.

**Table 1:** Study information for military recruits

Study	Country	Military service	Study design	Sampling timeframe	Duration of recruit training (weeks)	Injury case definition	Injury reporting (prospective/ retrospective)
Billings 2004 [40]	United States of America	Airforce	Cohort study	2002	6	Medical attention	Prospective
Blacker 2008 [11]	United Kingdom	Army	Cohort study	2003–2005	12	Medical attention	Retrospective
Booth 2006 [3]	Australia	Army	Cohort study	2003	6	Medical attention	Prospective
Brooks 2019 [41]	United States of America	Army	Cohort study	2007	9	Medical attention	Retrospective
Brushøj 2008 [42]	Denmark	Army	Randomised intervention trial	2004–2005	12	Medical attention	Prospective
Burley 2020 [43]	Australia	Army	Randomised intervention trial	Not reported	12	Medical attention	Prospective
Chassé 2020 [16]	Canada	Not reported	Cohort study	2016–2017	12	Medical attention	Prospective
Cowan 2011 [15, 37]	United States of America	Army	Cohort study	2005–2006	10	Medical attention	Retrospective
Cowan 2012 [44]	United States of America	Army	Cohort study	2005–2006	Not reported	Medical attention	Prospective
Dawson 2015 [45]	Australia	Army	Cohort study	2013	12	Medical attention	Prospective
Din 2016 [46]	Malaysia	Army	Cohort study	2013–2014	26	Medical attention	Prospective
Esterman 2005 [17]	Australia	Airforce	Randomised intervention trial	Not reported	10	Time-loss	Prospective
Everard 2018 [47]	Ireland	Army	Cohort study	Not reported	16	Time-loss	Prospective
Fallowfield 2020 [12]	United Kingdom	Airforce	Cohort study	2008	9	Time-loss	Prospective
Goodall 2013 [48]	Australia	Army	Randomised intervention trial	2007	12	Medical attention	Prospective
Hall 2017 [13]	United Kingdom	Army	Cohort study	2009–2011	14	Medical attention	Prospective
Hauschild 2018 [8]	United States of America	Army	Cohort study	2016	10	Medical attention	Prospective
Havenetidis 2011 [49]	Greece	Army	Cohort study	Not reported	7	Medical attention	Prospective
Havenetidis 2017 [50]	Greece	Army	Cohort study	Not reported	7	Medical attention	Prospective
Heagerty 2018 [18]	United Kingdom	Army	Cohort study	2012–2016	28	Medical attention	Prospective
Heller 2020 [4]	United Kingdom	Army	Cohort study	2016–2017	14	Time-loss	Prospective
Hofstetter 2012 [51]	Sweden	Army	Randomised intervention trial	2009–2010	7	Medical attention	Prospective
Jones 2017 [19]	United States of America	Army	Cohort study	2009 – 2012	10	Medical attention	Prospective
Kerr 2004 [52]	Ireland	Army	Cohort study	2000 – 2001	16	Medical attention	Prospective
Knapik 2006 [54]	United States of America	Army	Non-randomised intervention trial	2003	9	Medical attention	Prospective
Knapik 2010a [53]	United States of America	Airforce	Randomised intervention trial	2007	6	Medical attention	Prospective
Knapik 2010b [55]	United States of America	Marines	Randomised intervention trial	Not reported	12	Medical attention	Prospective
Mohammadi 2013 [56]	Iran	Army	Cohort Study	Not reported	8	Medical attention	Prospective
Müller-Schilling 2019 [5]	Germany	Army	Cohort Study	2012 – 2014	Not reported	Time-loss	Prospective
Munnoch 2007 [57]	United Kingdom	Marines	Cohort Study	2001 – 2002	32	Medical attention	Prospective
Nye 2016 [22]	United States of America	Airforce	Cohort study	2012–2014	8.5	Medical attention	Prospective
O’Connor 2011 [20]	United States of America	Marines	Cohort study	2009	16	Medical attention	Prospective
Orr 2020 [14]	Australia	Army	Cohort study	2006–2011	12	Medical attention	Prospective
	Australia	Army (Reserves)	Cohort study	2006–2011	4	Medical attention	Prospective
Roos 2015 [6]	Switzerland	Army	Non-randomised intervention trial	Not reported	21	Medical attention	Prospective
Schram 2019i [9]	Australia	Army	Cohort study	2012–2014	12	Medical attention	Retrospective
	Australia	Army (Reserves)	Cohort study	2012–2014	4	Medical attention	Retrospective
Sharma 2011 [7, 36]	United Kingdom	Army	Cohort study	Not reported	26	Medical attention	Prospective
Sharma 2015 [10, 35]	United Kingdom	Army	Cohort study	2006 – 2008	26	Medical attention	Prospective
Trone 2014 [58]	United States of America	Marines	Cohort study	2007	12	Medical attention	Prospective
Withnall 2006 [59]	United Kingdom	Airforce	Randomised intervention trial	2003 – 2004	9	Time-loss	Prospective
Wyss 2012 [61]	Switzerland	Army	Cohort study	Not reported	18	Medical attention	Prospective
Wyss 2014 [60]	Switzerland	Army	Cohort study	Not reported	18	Medical attention	Prospective

### Participant demographics 

The demographic details for each study are presented in Table 2. Overall, there were 451,782 recruits included across the 41 studies [3–20, 22, 35–37, 40–61]. The sample size for studies ranged from 22–184,670 recruits. Female representation in studies varied from 0–100% female inclusion. Seven studies did not report the sex of participating recruits. The mean age for the included studies ranged from 18–22 years.

**Table 2:** Demographic information for law enforcement recruits

Study	Sample size (n)	Total injured recruits (n)	Total injuries (n)	Total Sample sex split (% female)	Total Sample Age (years) – M (SD)	Total Sample Height (cm) – M (SD)	Total Sample Weight (kg) – M (SD)	Total Sample BMI (kg/m^2)^ – M (SD)	Injured Sample sex split (% female)	Injured Sample Age (years) – M (SD)	Injured Sample Height (cm) – M (SD)	Injured Sample Weight (kg) – M (SD)	Injured Sample BMI (kg/m^2)^ – M (SD)
Billings 2004 [40]	1210	-	846	18.5	18.4 (3.4)	-	-	23.9 (3.3)	28.3	-	-	-	-
Blacker 2008 [11]	13,417	793	793	11	20.5 (3.2)	175 (8)	70 (10)	23 (2)	32	-	-	-	-
Booth 2006 [3]	58	-	37	12	-	-	-	-	-	-	-	-	-
Brooks 2019 [41]	2000	820	-	27	-	-	-	-	62	-	-	-	-
Brushøj 2008 [42]	490	-	513	-	-	-	-	-	-	-	-	-	-
Burley 2020 [43]	69	-	17	24.3	-	-	-	-	-	-	-	-	-
Chassé 2020 [16]	6872	307	307	-	-	-	-	-	40.40	25.3 (6.9)	-	-	-
Cowan 2011 [15, 37]	7323	-	-	0	-	-	-	-	0	-	-	-	-
Cowan 2012 [44]	1568	1007	-	100	-	-	-	-	100	-	-	-	-
Dawson 2015 [45]	267	40	-	22.1	-	-	-	-	35	-	-	-	-
Din 2016 [46]	611	74	96	0	20.4 (1.9)	-	-	-	0	-	-	-	-
Esterman 2005 [17]	22	1	1	0	-	-	-	-	0	-	-	-	-
Everard 2018 [47]	132	-	28	0	22.4 (4.2)	177 (35)	74.5 (5.8)	-	0	-	-	-	-
Fallowfield 2020 [12]	1193	372	-	17	-	-	-	-	26.6	-	-	-	-
Goodall 2013 [48]	432	-	279	6	-	-	-	-	-	-	-	-	-
Hall 2017 [13]	3050	591	-	0	-	-	-	-	0	-	-	-	-
Hauschild 2018 [8]	106,367	33,005	65,025	20	-	-	-	-	30.3	-	-	-	-
Havenetidis 2011 [49]	233	66	191	0	20.1 (1.3)	177.6 (6.2)	77 (9.2)	-	-	-	-	-	-
Havenetidis 2017 [50]	268	86	86	0	20.4 (1.7)	177.7 (6.1)	79.3 (9.8)	-	0	20.4 (1.5)	177.7 (6.6)	80.1 (10.3)	-
Heagerty 2018 [18]	10,498	-	4101	-	-	-	-	-	-	-	-	-	-
Heller 2020 [4]	227	108	-	100	-	-	-	-	100	-	-	-	-
Hofstetter 2012 [51]	125	-	-	0	20.4 (1.2)	178 (10)	73.3 (9.1)	23.3 (2.6)	0	-	-	-	-
Jones 2017 [19]	184,670	39,328	-	23	Female = 22.3 (4.8), Male = 22.6 (4.9)	Female = 162 (6.4) Male = 176 (6.9)	Female = 61.8 (8.6), Male = 77.8 (13.2)	Female = 23.3(2.7), Male = 25.1 (3.7)	74.7	-	-	-	-
Kerr 2004 [52]	415	325	215	10.6	-	-	-	-	13	-	-	-	-
Knapik 2006 [54]	2072	-	-	43.3	-	-	-	-	-	-	-	-	-
Knapik 2010a [53]	-	-	-	27	-	-	-	-	-	-	-	-	-
Knapik 2010b [55]	689	-	-	37.3	-	-	-	-	-	-	-	-	-
Mohammadi 2013 [56]	50	8	8	0	21.4 (2.3)	174.5 (6.4)	73.1 (6.3)	24	0	21.9 (0.5)	175.1 (3.9)	72.9 (4.6)	23.7 (2.4)
Müller-Schilling 2019 [5]	774	255	397	-	20.5 (2.2)	179.6 (7.0)	75.9 (11.2)	23.5 (2.8)	-	-	-	-	-
Munnoch 2007 [57]	1115	166	166	0	20 (3)	-	-	-	0	20.1 (3)	176.8 (6.1)	73.5 (8)	23.4 (2.5)
Nye 2016 [22]	67,525	8448	11,673	21.6	-	-	-	-	33.9	-	-	-	-
O’Connor 2011 [20]	874	270	270	0	-	-	-	-	0	-	-	-	-
Orr 2020 [14]	12,077	4138	4138	8.2	-	-	-	-	-	-	-	-	-
	7692	1351	1351	13	-	-	-	-	-	-	-	-	-
Roos 2015 [6]	112	NR	66	0	20.54 (1.34)	177.76 (6.51)	71.86 (10.38)	22.71 (2.84)	0	-	-	-	-
Schram 2019 [9]	4452	1235	1235	-	-	-	-	-	-	-	-	-	-
	1630	150	150	-	-	-	-	-	-	-	-	-	-
Sharma 2011 [7, 36]	562	232	-	0	19.8 (2.3)	176.5 (6.8)	70.4 (9.7)	22.5 (2.5)	-	-	-	-	-
Sharma 2015 [10, 35]	6608	3215	3226	0	18.9 (2.3)	176.5 (7.8)	69 (9.7)	22.14 (2.5)	-	-	-	-	-
Trone 2014 [58]	1497	398		39.9	Female = 19.2 (2.0), Male = 20.7 (2.3)	Female = 163 (7), Male = 177 (7)	Female = 59.5 (7.5), Male = 77 (12.3)	-	34.5	-	-	-	-
Withnall 2006 [59]	401	86	86	22.7	19.8	175	70.3	22.8	-	-	-	-	-
Wyss 2012 [61]	459	-	320	-	-	-	-	-	-	-	-	-	-
Wyss 2014 [60]	1676	-	907	-	20.7 (1.2)	177.6 (6.3)	73.7 (10.6)	23.4 (3)	-	-	-	-	-

*Legend: n* Number, *M* Mean, *SD* Standard deviation, *cm* Centimetres, kg Kilograms, *BMI* Body mass index

### Assessment of heterogeneity

A substantial amount of missing data from participant demographics, as per Table 2, across studies precluded an assessment of heterogeneity. Furthermore, we considered the duration of recruit training sufficiently different between countries. Due to the substantial clinical diversity, data were not pooled between countries, and statistical heterogeneity was not calculated.

### Injury profiles

Thirty-five studies reported injury occurrence based on medical attention injuries [3, 6–11, 13–16, 18–20, 22, 35–37, 40–46, 48–58, 60, 61], whereas six studies reported based on time-loss injury case definitions [4, 5, 12, 17, 47, 59].

#### Injury frequency, proportion and prevalence

Five studies did not report the number of injured recruits or the number of total injuries. Most (*n* = 26;63%) studies reported the number of injured recruits, and the majority (*n* = 27;66%) of studies reported the number of injuries to recruits.

#### Prevalence of medical attention injuries 

Medical attention injuries were sustained by 22.8% of recruits (94,552 injured recruits within 414,498 recruits).

#### Prevalence of time-loss injuries

Time-loss injuries were sustained by 31.4% of recruits (822 injured recruits within 2,617 recruits).

#### Injury incidence and injury incidence rate 

Two studies were not included within the calculation of injury incidence as they did not indicate the recruit training duration [5, 44]. Five studies were not included in calculating injury incidence as they did not provide either the sample size or the number of injuries/ injured recruits [37, 51, 53–55].

#### Total injury incidence rates 

**Fig. 2:**
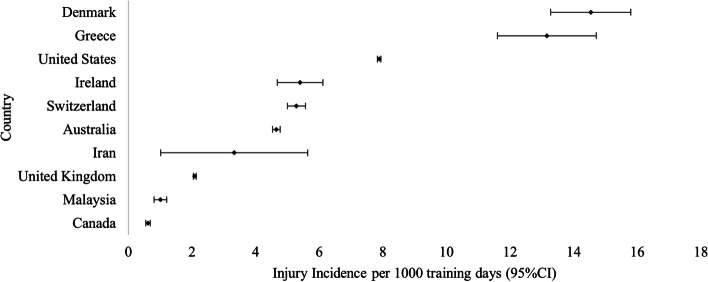
Pooled injury incidence rate per country

The injury incidence rate for medical attention injuries ranged from 0.62 injuries/1000 training days [16] to 19.52 injuries/1000 training days [49]. The injury incidence rate for recruits with a time-loss injury ranged from 0.75 injuries/1000 training days [17] to 3.97 injuries/1000 training days [59]. The complete layout of injury incidence rate, per country, is presented in Table 3 and the pooled injury incidence rate by country is also presented within Fig. 2.

**Table 3:** The injury incidence rates for recruits by country

Injury case definition	Country	Study	Incidence per 1000 training days (95%CI)
Medical Attention	Australia	Burley 2020	3.49 (1.80 to 5.05)
		Schram 2019	3.85 (3.64 to 4.07)
		Orr 2020	4.76 (4.61 to 4.90)
		Goodall 2013	8.97 (7.92 to 10.02)
		Booth 2006	17.7 (12.01 to 23.4)
		Pooled	4.65 (4.53 to 4.77)
	Australia (Reserves)	Schram 2019 (reserves)	3.83 (3.22 to 4.45)
		Orr 2020 (reserves)	7.31 (6.93 to 7.71)
		Pooled	6.71 (6.37 to 7.05)
	Canada	Chasse 2020	0.62 (0.55 to 0.69)
	Denmark	Brushøj 2008	14.54 (13.28 to 15.80)
	Greece	Havenetidis 2017	7.64 (6.02 to 9.26)
		Havenetidis 2011	19.52 (16.75 to 22.29)
		Pooled	13.16 (11.61 to 14.71)
	Iran	Mohammadi 2013	3.33 (1.02 to 5.64)
	Ireland	Kerr 2004	5.40 (4.68 to 6.12)
	Malaysia	Din 2016	1.01 (0.81 to 1.21)
	Switzerland	Roos 2015	4.68 (3.55 to 5.81)
		Wyss 2014	5.01 (4.68 to 5.33)
		Wyss 2012	6.46 (5.75 to 7.16)
		Pooled	5.28 (5.00 to 5.57)
	United Kingdom	Munnoch 2007	0.78 (0.66 to 0.89)
		Blacker 2008	0.82 (0.76 to 0.88)
		Heagerty 2018	2.33 (2.25 to 2.40)
		Sharma 2015	3.13 (3.02 to 3.24)
		Pooled	2.08 (2.04 to 2.13)
	United States of America	O’Connor 2011	3.22 (2.83 to 3.60)
		Nye 2016	3.39 (3.33 to 3.45)
		Cowan 2011	8.99 (8.76 to 9.22)
		Hauschild 2018	10.19 (10.11 to 10.27)
		Billings 2004	19.42 (18.11 to 20.73)
		Pooled	7.89 (7.83 to 7.94)
Time-loss	Australia	Esterman 2005	0.75 (0.00 to 2.24)
	Ireland	Everard 2018	2.21 (1.39 to 3.03)
	United Kingdom	Withnall 2006	3.13 to 4.81)

#### Influence of training duration in injury incidence rate

The injury incidence rate appeared to be associated with the duration of recruit training. The generalised linear model (Appendix F) demonstrated that every week longer in duration a recruit training protocol, there appeared to be an associated reduction of (95%CI = -0.616 to -0.131) in the injury incidence rate (*p* = 0.003). The AIC was 160, demonstrated adequate model fit.

### Assessment of quality in included studies

The overall quality for 26 studies (63.4%) was classified as high and 15 studies (36.6%) were classified as low quality (Appendix G). Twenty-five studies (61%) had an appropriate sample frame. Thirty-one studies (76%) sampled participants appropriately. Twenty-five studies (61%) had an appropriate sample size. Fourteen studies (34.1%) adequately described the participants and settings. Thirty-one studies (76%) had sufficient coverage of the included sample within analyses. Thirty-four (83%) used valid measures to identify the condition. Thirty-four (83%) used reliable methods to quantify the condition (typically the international classification of disease codes). Thirty-seven studies (90%) had adequate response rates (typically due to studies being retrospective audits of medical records).

### Assessment of the certainty of the body of evidence

Injury incidence rates were calculated using the number of injuries and the duration of the recruit training program. However, the certainty for the injury incidence rates we calculated were judged to be low, suggesting the true incidence rates might be markedly different from the estimated rate. The certainty of the evidence was downgraded due to several reasons. Firstly, over one-third of studies were considered low quality and included less than 200 injuries. Secondly, due to indirectness based on 65.9% of studies did not adequately report the participant demographics and setting, with no studies detailing the physical training interventions. Lastly, due to inconsistency, based upon the poor overlap of the 95% confidence intervals of the injury incidence rate assessed per country.

## Discussion

Injuries are prevalent across all counties in military recruit populations, with our results indicating that one-in-four recruits seek medical attention due to injury in one training period. These injury outcomes are far higher than recruits of other tactical populations, such as law enforcement [1, 31]. Military recruit injury incidence rates published within the last five years were between 0.62–10.19 injuries/1000 days and may be overall trending less than those studies published between 2000–2017, with injury rates over this period between 0.78–19.52/1000 training days, however this seems unlikely. Unfortunately, statistical analysis to confirm this is not possible with the available data. These injury incidence rates remain a concern as previous injury is a known risk factor for future injury in the military [62]. Consequently, injuries sustained early in a recruit’s career may lead to further problems once qualified [62]. Injuries sustained as recruits (e.g., Anterior Cruciate Ligament rupture) may also have lifelong implications to function and quality of life [63, 64]. Thus, considering the current high injury rates and vulnerability for future injury, military recruits remain a significant priority military subgroup for injury prevention.

The duration of recruit training was identified as being associated with the recruit injury incidence rate. Our results demonstrate that longer recruit training programs are associated with a lower injury incidence rate. This appears reasonable when considering longer programs will be better able to spread physical training over the duration of the program, as opposed to needing all fitness to be completed in a short window. By having a longer program, and reducing large increases in training volume, recruits are less likely to become acutely overloaded and suffer an injury [65]. However, these results must be interpreted with caution given many other factors contribute to the injury incidence rate that were unable to be controlled for within our model.

Previous literature has identified that female sex is a risk factor for injury [62]. This appears to be consistent with our research. While more males were injured than females overall, a higher proportion of females sustained injuries when accounting for the number of females included within the respective study. However, females were represented in approximately half the studies identified, of which only 11 studies reported injury incidence by sex. We recommend future research reports total injury rates along with injury rates by sex to identify at-risk groups clearly. Only three studies reported on height, weight, and BMI, thus it is unclear if such biometric information is associated with higher injury rates in recruits. Data related to age and BMI are important to include when describing study populations, given their influence on injury rates [66–69]. A recent meta-analysis has established high BMI as a predictor for injury in general military populations, along with lower fitness standards. Multiple countries have highlighted the growing prevalence of obesity in the military [5, 70–72]. Concurrently, some military organisations are lowering entry fitness standards required to enlist in military service in specific roles. We recommend, where possible, that future surveillance studies consider recording these evidence-based risk factors to determine the relationship between BMI, fitness and injury risk in military recruits to identify potential injury patterns associated with such changes. Such information is essential to inform future policies related to recruitment health and fitness standards and injury prevention.

These injury frequency findings are likely to underestimate the actual burden of injury in military recruits due to the methods used to collect injury data. All studies used data based on recruits engaging in military health systems. It is known that many recruits will purposely avoid doing for various reasons [73, 74], such as fear of affecting career aspirations. Research in combat units suggests that approximately half of the personnel do not report their injuries, and thus a significant number of injuries are not recorded by surveillance systems [75]. Injury underreporting compromises the accuracy of surveillance and limits the proper identification of prevention prioritises. Injury consensus guidelines in the military recommend alternative surveillance methods, such as anonymous surveys where personnel can report injury data without fear of repercussions [26]. Previous literature differs on whether female military members are more likely to engage with military healthcare systems when injured compared to their male counterparts [62]. Such injury reporting behaviours could influence epidemiological outcomes when using medical attention data to calculate injury rates and should be considered when considering sex-related subgroup injury risks [62].

There appeared to be considerable clinical diversity and heterogeneity between studies in the studies identified, even with studies from the same country, service or authors, as seen by the minimal overlap of the 95% confidence intervals of the injury incidence rate between studies. This heterogeneity may arise from differences within the basic training program or different samples. It is not possible to determine the influence of these given the details of the training programs are not reported, and few studies report extensive demographic information of their recruits [9, 14, 18, 42, 43, 48, 53–55, 61]. The year difference between studies may also influence the injury incidence rate as the training program, and injury prevention interventions would likely change over time. Again, this is difficult to quantify from the current literature.

### Limitations

Due to a lack of reporting of detailed exposure metrics (e.g., hours of physical activity within the recruit training program) within most studies, one week of recruit training was assumed to represent six training exposure days when calculating the injury incidence rate. We are aware that some military training programs may have fewer or more training days in one week than this. Therefore, our exposure calculations may not be entirely accurate, and exposure is likely to be marginally overestimated as we could not remove training days post-injury from our analyses. If a study did not report whether injuries were based on the total number of injuries or the number of injured recruits, for the purposes of calculating the injury incidence, we assumed they reported the number of injured recruits. However, we contend that injury rates during training programs from > 20 years ago would provide limited evidence applicable to current training approaches. While multiple authors completed screening and extraction, a single author (MCM) completed database searches, which may lead to bias. However, the author has completed search strategies for multiple systematic reviews [31, 76–78] and the influence of this was deemed to be negligible.

## Conclusion

This review identified 41 studies that reported the injury epidemiology frequency of military recruits. Injuries are prevalent in military recruits, with up to one-in-four of recruits seeking medical assistance for injury in one training period. Such findings reinforce that military recruits are an organisational priority for injury prevention. There may be a pattern demonstrating improving injury rates within recent years; however, the overall certainty for the accuracy of our injury frequency results is low due to our study quality assessment findings. Future research should apply military-specific recommended injury surveillance guidelines to improve future surveillance accuracy and comparison. Further knowledge needs to be established about specific basic training activities associated with injury and injury risk associated with BMI.

### Supplementary Information

**Additional file 1:** **Appendix A. **Systematic Review Search Strategy. **Appendix B.** Search Strategy Documentation. **Appendix C**. The quality assessment checklist and criteria used to assess the quality of individual studies. **Appendix D. **Recruit injury incidence rates (excluding recurrent/ subsequent injuries). The injury incidence rate for recruits (excluding recurrent/ subsequent injuries) with a medical attention injury ranged from 0.62 injured recruits per 1000 training days [1] to 8.12 injured recruits per 1000 training days. [2] The injury incidence rate for recruits with a time-loss injury (excluding recurrent/ subsequent injuries) ranged from 0.76 injured recruits per 1000 training days [3] to 5.77 injured recruits per 1000 training days. [4]. **Appendix E.** Study funding sources. **Appendix F.** Generalised linear model of study level data, assessing the association between the medical attention injury incidence rate (95% CI), and the duration of the recruit training program (weeks). **Appendix G.** Quality of include studies

## Data Availability

All data generated or analysed during this study are included in this published article and supplementary appendices.

## References

[1] Merrick N, Hart NH, Mosler AB, Allen G, Murphy MC. The injury profiles of police officer recruits undergoing basic physical training: a prospective cohort study. J Occup Rehabil. 2023;33(1):170–8. 10.1007/s10926-022-10059-2 PMC10025230 35917080

[2] Dijksma I, Sharma J, Gabbett TJ (2021). Training load monitoring and injury prevention in military recruits: considerations for preparing soldiers to fight sustainably. Strength Condition J.

[3] Booth CK, Probert B, Forbes-Ewan C, Coad RA (2006). Australian army recruits in training display symptoms of overtraining. Mil Med.

[4] Heller R, Stammers H. Running to breaking point? The relationship between 1.5-mile run time and injury risk in female recruits during British Army basic training. BMJ Mil Health. 2020;166(E):e3–e7. 10.1136/jramc-2018-001012 30755471

[5] Müller-Schilling L, Gundlach N, Böckelmann I, Sammito S (2019). Physical fitness as a risk factor for injuries and excessive stress symptoms during basic military training. Int Arch Occup Environ Health.

[6] Roos L, Boesch M, Sefidan S, Frey F, Mäder U, Annen H (2015). Adapted marching distances and physical training decrease recruits' injuries and attrition. Mil Med.

[7] Sharma J, Golby J, Greeves J, Spears IR (2011). Biomechanical and lifestyle risk factors for medial tibia stress syndrome in army recruits: a prospective study. Gait Posture.

[8] Hauschild VD, Lee T, Barnes S, Forrest L, Hauret K, Jones BH (2018). The etiology of injuries in us army initial entry training. US Army Med Dep J.

[9] Schram B, Pope R, Orr R (2019). Injuries in Australian Army full-time and part-time personnel undertaking basic training. BMC Musculoskelet Disord.

[10] Sharma J, Greeves JP, Byers M, Bennett AN, Spears IR (2015). Musculoskeletal injuries in British Army recruits: a prospective study of diagnosis-specific incidence and rehabilitation times. BMC Musculoskelet Disord.

[11] Blacker SD, Wilkinson DM, Bilzon JL, Rayson MP (2008). Risk factors for training injuries among British Army recruits. Mil Med.

[12] Fallowfield JL, Leiper RG, Shaw AM, Whittamore DR, Lanham-New SA, Allsopp AJ (2020). Risk of injury in royal air force training: does sex really matter?. Mil Med.

[13] Hall LJ (2017). Relationship between 1.5-mile run time, injury risk and training outcome in British Army recruits. J R Army Med Corps.

[14] Orr RM, Cohen BS, Allison SC, Bulathsinhala L, Zambraski EJ, Jaffrey M (2020). Models to predict injury, physical fitness failure and attrition in recruit training: a retrospective cohort study. Mil Med Res.

[15] Bedno SA, Cowan DN, Urban N, Niebuhr DW (2013). Effect of pre-accession physical fitness on training injuries among US Army recruits. Work (Reading, Mass).

[16] Chassé E, Laroche MA, Dufour CA, Guimond R, Lalonde F (2020). Association between musculoskeletal injuries and the Canadian armed forces physical employment standard proxy in Canadian military recruits. Mil Med.

[17] Esterman A, Pilotto L. Foot shape and its effect on functioning in Royal Australian Air Force recruits. Part 1: Prospective cohort study. Mil Med. 2005;170(7):623–8. 10.7205/milmed.170.7.623 16130646

[18] Heagerty R, Sharma J, Cayton J, Goodwin N (2018). Retrospective analysis of four-year injury data from the Infantry training Centre. Catterick J R Army Med Corps.

[19] Jones BH, Hauret KG, Dye SK, Hauschild VD, Rossi SP, Richardson MD (2017). Impact of physical fitness and body composition on injury risk among active young adults: a study of army trainees. J Sci Med Sport.

[20] O'Connor FG, Deuster PA, Davis J, Pappas CG, Knapik JJ (2011). Functional movement screening: predicting injuries in officer candidates. Med Sci Sports Exerc.

[21] Dijksma I, Zimmermann WO, Hertenberg EJ, Lucas C, Stuiver MM. One out of four recruits drops out from elite military training due to musculoskeletal injuries in the Netherlands Armed Forces. BMJ Mil Health. 2022;168(2):136–40. 10.1136/bmjmilitary-2020-001420 PMC8961760 32139408

[22] Nye NS, Pawlak MT, Webber BJ, Tchandja JN, Milner MR (2016). Description and rate of Musculoskeletal injuries in air force basic military trainees, 2012–2014. J Athl Train.

[23] Lovalekar M, Sharp MA, Billing DC, Drain JR, Nindl BC, Zambraski EJ (2018). International consensus on military research priorities and gaps - survey results from the 4th international congress on soldiers’ physical performance. J Sci Med Sport.

[24] Stannard J, Fortington L (2021). Musculoskeletal injury in military special operations forces: a systematic review. BMJ Mil Health.

[25] Bahr R, Clarsen B, Derman W, Dvorak J, Emery CA, Finch CF (2020). International Olympic Committee consensus statement: methods for recording and reporting of epidemiological data on injury and illness in sport 2020 (including STROBE Extension for Sport Injury and Illness Surveillance (STROBE-SIIS)). Br J Sports Med.

[26] Stannard J, Finch CF, Fortington LV (2022). Improving musculoskeletal injury surveillance methods in special operation forces: a Delphi consensus study. PLOS Global Public Health.

[27] Page MJ, McKenzie JE, Bossuyt PM, Boutron I, Hoffmann TC, Mulrow CD (2021). The PRISMA 2020 statement: an updated guideline for reporting systematic reviews. BMJ (Clinical research ed).

[28] Morrison A, Polisena J, Husereau D, Moulton K, Clark M, Fiander M (2012). The effect of English-language restriction on systematic review-based meta-analyses: a systematic review of empirical studies. Int J Technol Assess Health Care.

[29] Nindl BC, Jones BH, Van Arsdale SJ, Kelly K, Kraemer WJ (2016). Operational physical performance and fitness in military women: physiological, musculoskeletal injury, and optimized physical training considerations for successfully integrating women into combat-centric military occupations. Military Medicine.

[30] Vaara JP, Groeller H, Drain J, Kyröläinen H, Pihlainen K, Ojanen T, et al. Physical training considerations for optimizing performance in essential military tasks. Eur J Sport Sci. 2022;22(1):43–57. 10.1080/17461391.2021.1930193 34006204

[31] Murphy M, George H, Naqi M, Owen P, Chivers P, Hart NH. Musculoskeletal injury epidemiology in law enforcement and firefighter recruits during physical training: a systematic review. BMJ Open Sport Exerc Med. 2022;8(1):e001289. 10.1136/bmjsem-2021-001289 PMC8889355 35309374

[32] Sammito S, Hadzic V, Karakolis T, Kelly KR, Proctor SP, Stepens A (2021). Risk factors for musculoskeletal injuries in the military: a qualitative systematic review of the literature from the past two decades and a new prioritizing injury model. Mil Med Res.

[33] Molloy JM (2016). Factors influencing running-related musculoskeletal injury risk among U. S. military recruits. Military Medicine.

[34] Liberati A, Altman DG, Tetzlaff J, Mulrow C, Gotzsche PC, Ioannidis JP (2009). The PRISMA statement for reporting systematic reviews and meta-analyses of studies that evaluate healthcare interventions: explanation and elaboration. BMJ (Clinical research ed).

[35] Sharma J, Dixon J, Dalal S, Heagerty R, Spears I (2017). Musculoskeletal injuries in British Army recruits: a prospective study of incidence in different Infantry Regiments. J R Army Med Corps.

[36] Sharma J, Heagerty R, Dalal S, Banerjee B, Booker T (2019). Risk factors associated with musculoskeletal injury: a prospective study of British infantry recruits. Curr Rheumatol Rev.

[37] Cowan DN, Bedno SA, Urban N, Yi B, Niebuhr DW (2011). Musculoskeletal injuries among overweight army trainees: incidence and health care utilization. Occupational medicine (Oxford, England).

[38] Dechartres A, Trinquart L, Boutron I, Ravaud P (2013). Influence of trial sample size on treatment effect estimates: meta-epidemiological study. BMJ (Clinical research ed).

[39] Morgan RL, Thayer KA, Bero L, Bruce N, Falck-Ytter Y, Ghersi D (2016). GRADE: assessing the quality of evidence in environmental and occupational health. Environ Int.

[40] Billings CE (2004). Epidemiology of injuries and illnesses during the United States air force academy 2002 basic cadet training program: documenting the need for prevention. Mil Med.

[41] Brooks RD, Grier T, Dada EO, Jones BH (2019). The combined effect of cigarette smoking and fitness on injury risk in men and women. Nicotine Tobacco Res: Official J Soc Res Nicotine Tobacco.

[42] Brushøj C, Larsen K, Albrecht-Beste E, Nielsen MB, Løye F, Hölmich P (2008). Prevention of overuse injuries by a concurrent exercise program in subjects exposed to an increase in training load: a randomized controlled trial of 1020 army recruits. Am J Sports Med.

[43] Burley SD, Drain JR, Sampson JA, Nindl BC, Groeller H (2020). Effect of a novel low volume, high intensity concurrent training regimen on recruit fitness and resilience. J Sci Med Sport.

[44] Cowan DN, Bedno SA, Urban N, Lee DS, Niebuhr DW (2012). Step test performance and risk of stress fractures among female army trainees. Am J Prev Med.

[45] Dawson GME, Broad R, Orr RM (2015). The impact of a lengthened Australian Army recruit training course on recruit injuries. J Military Veterans’ Health.

[46] Din FHM, Rampal S, Muslan MA, Hoe VCW (2016). Association between pain catastrophising and musculoskeletal disorders is modified by past injuries in Malaysian military recruits. Occup Environ Med.

[47] Everard E, Lyons M, Harrison AJ (2018). Examining the association of injury with the functional movement screen and landing error scoring system in military recruits undergoing 16 weeks of introductory fitness training. J Sci Med Sport.

[48] Goodall RL, Pope RP, Coyle JA, Neumayer R (2013). Balance and agility training does not always decrease lower limb injury risks: a cluster-randomised controlled trial. Int J Inj Contr Saf Promot.

[49] Havenetidis K, Kardaris D, Paxinos T (2011). Profiles of musculoskeletal injuries among Greek army officer cadets during basic combat training. Mil Med.

[50] Havenetidis K, Paxinos T, Kardaris D, Bissas A (2017). Prognostic potential of body composition indices in detecting risk of musculoskeletal injury in army officer cadet profiles. Phys Sportsmed.

[51] Hofstetter MC, Mäder U, Wyss T (2012). Effects of a 7-week outdoor circuit training program on Swiss army recruits. J Strength Cond Res.

[52] Kerr GM (2004). Injuries sustained by recruits during basic training in Irish Army. Ir Med J.

[53] Knapik JJ, Brosch LC, Venuto M, Swedler DI, Bullock SH, Gaines LS (2010). Effect on injuries of assigning shoes based on foot shape in air force basic training. Am J Prev Med.

[54] Knapik JJ, Darakjy S, Hauret KG, Canada S, Scott S, Rieger W (2006). Increasing the physical fitness of low-fit recruits before basic combat training: an evaluation of fitness, injuries, and training outcomes. Mil Med.

[55] Knapik JJ, Trone DW, Swedler DI, Villasenor A, Bullock SH, Schmied E (2010). Injury reduction effectiveness of assigning running shoes based on plantar shape in Marine Corps basic training. Am J Sports Med.

[56] Mohammadi F, Azma K, Naseh I, Emadifard R, Etemadi Y (2013). Military exercises, knee and ankle joint position sense, and injury in male conscripts: a pilot study. J Athl Train.

[57] Munnoch K, Bridger RS (2007). Smoking and injury in Royal Marines' training. Occupational medicine (Oxford, England).

[58] Trone DW, Cipriani DJ, Raman R, Wingard DL, Shaffer RA, Macera CA. Self-reported smoking and musculoskeletal overuse injury among male and female U.S. Marine Corps recruits. Military Med. 2014;179(7):735–43. 10.7205/MILMED-D-13-00516 25003858

[59] Withnall R, Eastaugh J, Freemantle N (2006). Do shock absorbing insoles in recruits undertaking high levels of physical activity reduce lower limb injury? A randomized controlled trial. J R Soc Med.

[60] Wyss T, Roos L, Hofstetter MC, Frey F, Mäder U (2014). Impact of training patterns on injury incidences in 12 Swiss Army basic military training schools. Mil Med.

[61] Wyss T, Von Vigier RO, Frey F, Mäder U (2012). The Swiss Army physical fitness test battery predicts risk of overuse injuries among recruits. J Sports Med Phys Fitness.

[62] Rhon DI, Molloy JM, Monnier A, Hando BR, Newman PM (2022). Much work remains to reach consensus on musculoskeletal injury risk in military service members: A systematic review with meta-analysis. Eur J Sport Sci.

[63] Maffulli N, Longo UG, Gougoulias N, Loppini M, Denaro V (2010). Long-term health outcomes of youth sports injuries. Br J Sports Med.

[64] Culvenor AG, Collins NJ, Guermazi A, Cook JL, Vicenzino B, Whitehead TS (2016). Early patellofemoral osteoarthritis features one year after anterior cruciate ligament reconstruction: symptoms and quality of life at three years. Arthritis Care Res.

[65] Gabbett TJ (2016). The training—injury prevention paradox: should athletes be training smarter <em>and</em> harder?. Br J Sports Med.

[66] Freckleton G, Pizzari T (2013). Risk factors for hamstring muscle strain injury in sport: a systematic review and meta-analysis. Br J Sports Med.

[67] Green B, Pizzari T (2017). Calf muscle strain injuries in sport: a systematic review of risk factors for injury. Br J Sports Med.

[68] Paulis WD, Silva S, Koes BW, van Middelkoop M (2014). Overweight and obesity are associated with musculoskeletal complaints as early as childhood: a systematic review. Obes Rev.

[69] Smith KL, Weir PL, Till K, Romann M, Cobley S (2018). Relative age effects across and within female sport contexts: a systematic review and meta-analysis. Sports Med.

[70] Quertier D, Goudard Y, Goin G, Régis-Marigny L, Sockeel P, Dutour A (2022). Overweight and obesity in the french army. Mil Med.

[71] Salimi Y, Taghdir M, Sepandi M, Karimi Zarchi AA (2019). The prevalence of overweight and obesity among Iranian military personnel: a systematic review and meta-analysis. BMC Public Health.

[72] Smith TJ, Marriott BP, Dotson L, Bathalon GP, Funderburk L, White A, et al. Overweight and obesity in military personnel: sociodemographic predictors. Obesity. 2012;20(7):1534–8. 10.1038/oby.2012.25 22314620

[73] Cohen BS, Pacheco BM, Foulis SA, Canino MC, Redmond JE, Westrick RB (2019). Surveyed reasons for not seeking medical care regarding musculoskeletal injury symptoms in US army trainees. Mil Med.

[74] Rawlins MLW, Johnson BR, Register-Mihalik JK, DeAngelis K, Schmidt JD, D'Lauro CJ (2020). United States air force academy cadets’ perceived costs of concussion disclosure. Mil Med.

[75] Smith L, Westrick R, Sauers S, Cooper A, Scofield D, Claro P (2016). Underreporting of musculoskeletal injuries in the US army: findings from an infantry brigade combat team survey study. Sports Health.

[76] Murphy MC, Travers MJ, Chivers P, Debenham JR, Docking SI, Rio EK (2019). Efficacy of heavy eccentric calf training for treating mid-portion Achilles tendinopathy: a systematic review and meta-analysis. Br J Sports Med.

[77] Murphy M, Travers MJ, Gibson W, Chivers P, Debenham J, Docking S (2018). The rate of improvement of pain and function in mid-portion Achilles tendinopathy with loading protocols: a systematic review and longitudinal meta-analysis. Sports Med.

[78] Harris SA, Dempsey AR, Mackie K, King D, Hecimovich M, Murphy MC. Do Sideline Tests of Vestibular and Oculomotor Function Accurately Diagnose Sports-Related Concussion in Adults? A Systematic Review and Meta-analysis. Am J Sports Med. 2022;50(9):2542–51. 10.1177/03635465211027946 34432554

