# Supplementary material for: Epidemiology of musculoskeletal injury in military recruits: a systematic review and meta-analysis
Source: BMC Sports Sci Med Rehabil. 2023 Oct 28;15:144. doi: 10.1186/s13102-023-00755-8 (PMC10612319; doi:10.1186/s13102-023-00755-8)
Supplement: Supplementary file 1 — Additional file 1: Appendix A. Systematic Review Search Strategy. Appendix B. Search Strategy Documentation. Appendix C. The quality assessment checklist and criteria used to assess the quality of individual studies. Appendix D. Recruit injury incidence rates (excluding recurrent/ subsequent injuries). The injury incidence rate for recruits (excluding recurrent/ subsequent injuries) with a medical attention injury ranged from 0.62 injured recruits per 1000 training days [1] to 8.12 injured recruits per 1000 training days. [2] The injury incidence rate for recruits with a time-loss injury (excluding recurrent/ subsequent injuries) ranged from 0.76 injured recruits per 1000 training days [3] to 5.77 injured recruits per 1000 training days. [4]. Appendix E. Study funding sources. Appendix F. Generalised linear model of study level data, assessing the association between the medical attention injury incidence rate (95% CI), and the duration of the recruit training program (weeks). Appendix G. Quality of include studies [file 13102_2023_755_MOESM1_ESM.docx]

**Appendix A.** Systematic Review Search Strategy

| **Number** | **Combiners** | **Terms** |
| --- | --- | --- |
| 1 | Problem of Interest | Injur* |
| 2 | Participants | Recruit* OR trainee* |
| 3 | Participants | Defence OR defense OR army OR military OR armed forces OR navy OR air force OR marine* OR coast guard OR national guard OR submariner OR sailor OR soldier |
| 4 | Exclusion | review OR meta-analysis |
| 5 |  | #1 AND #2 AND #3 NOT #4 |
|  | Limitations | Peer-reviewed, human, English language, |

**Appendix B.** Search Strategy Documentation

|  |  | | | | |
| --- | --- | --- | --- | --- | --- |
| Source: | Date of search | Search strategy used (keywords & Boolean) | Search Limits or filters (e.g. dates, language) | # results found | Comments |
| PUBMED | 05/05/2021 | (((Injur*) AND (Recruit* OR trainee*)) AND (Defence OR defense OR army OR military OR armed forces OR navy OR air force OR marine* OR coast guard OR national guard OR submariner OR sailor OR soldier)) NOT (review OR meta-analysis) | Human, English | 868 | Exported to End Note |
| CINAHL (Full Text) | 05/05/2021 | (((Injur*) AND (Recruit* OR trainee*)) AND (Defence OR defense OR army OR military OR armed forces OR navy OR air force OR marine* OR coast guard OR national guard OR submariner OR sailor OR soldier)) NOT (review OR meta-analysis) | Peer reviewed, human, English | 562 | Exported to End Note |
| CENTRAL | 05/05/2021 | (((Injury) AND (Recruit OR trainee)) AND (Defence OR defense OR army OR military OR armed forces OR navy OR air force OR marine OR coast guard OR national guard OR submariner OR sailor OR soldier)) | Word variations, trials | 702 | Exported to End Note |
| SportsDISCUS | 05/05/2021 | (((Injur*) AND (Recruit* OR trainee*)) AND (Defence OR defense OR army OR military OR armed forces OR navy OR air force OR marine* OR coast guard OR national guard OR submariner OR sailor OR soldier)) NOT (review OR meta-analysis) | Peer-reviewed, English | 913 | Exported to End Note |
| Web of Science | 05/05/2021 | (((AB= Injur* AND AB=(Recruit* OR trainee*) AND AB=(Defence OR defense OR army OR military OR armed forces OR navy OR air force OR marine* OR coast guard OR national guard OR submariner OR sailor OR soldier)) NOT ALL=(review OR meta-analysis))) | English, article | 682 | Exported to End Note |
| Key Journals |  |  |  |  | N/A |
| TOTAL | | | | | 3727 |

**Appendix C**. The quality assessment checklist and criteria used to assess the quality of individual studies.

| Checklist item | Marking criteria |
| --- | --- |
| 1. Was the sample frame appropriate to address the target population? | Studies were classified as high quality if the study was a census, database, or large population-based survey. |
| 1. Were study recruits sampled in an appropriate way? | Studies were classified as high quality if all recruits from the sample frame were eligible to be included. |
| 1. Was the sample size adequate | Studies were classified as high quality if they included greater than 500 recruits. |
| 1. Were the study subjects and the setting described in detail? | Studies that reported recruit age, sex and training program duration were classified as high quality |
| 1. Was the data analysis conducted with sufficient coverage of the identified sample? | Studies were classified as high quality if the study reported a similar response rate across demographic sub-groups. |
| 1. Were valid methods used for the identification of the condition? | Studies were classified as high quality if an injury diagnosis was obtained via a health care provider, e.g., medical doctor or physical therapist. |
| 1. Was the condition measured in a standard, reliable way for all recruits? | Studies were classified as high quality if the same healthcare provider classified all recruits within the sample or a standard template recording form was used. |
| 1. Was there appropriate statistical analysis? | Studies were not assessed against this item as this item was considered not applicable within our research. |
| 1. Was the response rate adequate, and if not, was the low response rate managed appropriately? | Studies were classified as high quality if the response rate was higher than 50% or less than 50% and managed appropriately (e.g., responses were assessed to ensure they were representative of the sample, such as ensuring the sex split of responders was comparable to the sample. |

**Appendix D.** Recruit injury incidence rates (excluding recurrent/ subsequent injuries). The injury incidence rate for recruits (excluding recurrent/ subsequent injuries) with a medical attention injury ranged from 0.62 injured recruits per 1000 training days[1] to 8.12 injured recruits per 1000 training days.[2] The injury incidence rate for recruits with a time-loss injury (excluding recurrent/ subsequent injuries) ranged from 0.76 injured recruits per 1000 training days[3] to 5.77 injured recruits per 1000 training days.[4]

| Injury case definition | Country | Study | Incidence per 1000 training days (95%CI) |
| --- | --- | --- | --- |
| Medical attention | Australia | Dawson 2015 | 2.08 (1.44 to 2.73) |
|  |  | Schram 2019 | 3.83 (3.22 to 4.45) |
|  |  | Orr 2020 | 4.76 (4.61 to 4.90) |
|  | Australia (reserves) | Schram 2019 (reserves) | 3.85 (3.64 to 4.07) |
|  |  | Orr 2020 (reserves) | 7.32 (6.93 to 7.71) |
|  | Canada | Chasse 2020 | 0.62 (0.55 to 0.69) |
|  | Greece | Havenetidis 2011 | 6.74 (5.12 to 8.37) |
|  |  | Havenetidis 2017 | 7.64 (6.03 to 9.26) |
|  | Iran | Mohammadi 2013 | 3.33 (1.02 to 5.64) |
|  | Ireland | Kerr 2004 | 8.12 (7.27 to 9.04) |
|  | Malaysia | Din 2016 | 0.78 (0.60 to 0.95) |
|  | United Kingdom | Munnoch 2007 | 0.78 (0.66 to 0.89) |
|  |  | Sharma 2011 | 2.65 (2.31 to 2.99) |
|  |  | Sharma 2015 | 3.12 (3.01 to 3.23) |
|  |  | Blacker 2008 | 0.82 (0.76 to 0.89) |
|  |  | Hall 2017 | 2.31 (2.12 to 2.49) |
|  |  | Heller 2020 | 5.66 (4.60 to 6.73) |
|  | United States of America | O’Connor 2011 | 3.22 (2.83 to 3.61) |
|  |  | Jones 2017 | 3.55 (3.51 to 3.58) |
|  |  | Trone 2014 | 3.69 (3.33 to 4.06) |
|  |  | Brooks 2019 | 7.59 (7.07 to 8.11) |
|  |  | Nye 2016 | 2.45 (2.40 to 2.51) |
|  |  | Cowan 2011 | 3.67 (3.52 to 3.81) |
|  |  | Hauschild 2018 | 5.17 (5.12 to 5.23) |
| Time-loss | Australia | Esterman 2005 | 0.76 (0.00 to 2.24) |
|  | United Kingdom | Withnall 2006 | 3.97 (3.12 to 4.81) |
|  |  | Fallowfield 2020 | 5.77 (5.18 to 6.36) |

**Appendix E**. Study funding sources

| Study | Funding source |
| --- | --- |
| Billings 2004[5] | US Air force academy |
| Blacker 2008[6] | Not reported |
| Booth 2006[7] | Not reported |
| Brooks 2019[8] | This investigation was supported in part by an appointment to the Postgraduate Research Participation Program at the US Army Public Health Center, administered by the Oak Ridge Institute for Science and Education, through an interagency agreement between the US Department of Energy and USAPHC. This research did not receive any specific grant from funding agen- cies in the public, commercial, or not-for-profit sectors |
| Brushøj 2008[9] | None Disclosed |
| Burley 2020[10] | Not reported |
| Chassé 2020[1] | Not reported |
| Cowan 2011[11, 12] | United States Military Entrance Processing Command; Army Accession Command (MIPR9DDATFTM04). |
| Cowan 2012[13] | U.S. Army Accession Command and the U.S. Army National Guard Bureau |
| Dawson 2015[14] | Not reported |
| Din 2016[15] | FHMD was supported by the Malaysian Armed Forces Higher Education's Academic Training Scheme. VCWH and SR were supported by the Ministry of Education High Impact Research Grant (H-20001-00-E2000069) |
| Esterman 2005[3] | Not reported |
| Everard 2018[16] | There was no financial assistance or gain for anyone associated with or conducting this study. |
| Fallowfield 2020[4] | This study was supported financially by the United Kingdom Ministry of Defence, and by the Arthritis Research United Kingdom Centre for Sport, Exercise and Osteoarthritis (Grant reference 21595) |
| Goodall 2013[17] | There was no external financial support for this project |
| Hall 2017[18] | The British Army Postgraduate Deanery funded my MSc Sports and Exercise Medicine at University of Wales Institute Cardiff, Cyncoed Campus, Cyncoed Road, Cardiff, CF23 6XD. I was not given any other additional funding from external sources to conduct the study. |
| Hauschild 2018[19] | Not reported |
| Havenetidis 2011[20] | This study was funded by Greek ministry of defence |
| Havenetidis 2017[21] | This paper was not funded |
| Heagerty 2018[22] | Not reported |
| Heller 2020[23] | Funding Both authors are employed by the British Army. No additional funding from external sources was given to conduct the study |
| Hofstetter 2012[24] | No funding received for this work |
| Jones 2017[25] | None Disclosed |
| Kerr 2004[2] | None Disclosed |
| Knapik 2006[26] | None Disclosed |
| Knapik 2010a[27] | None Disclosed |
| Knapik 2010b[28] | This research was supported by the Naval Health Research Center |
| Mohammadi 2013[29] | ND |
| Müller-Schilling 2019[30] | ND |
| Munnoch 2007[31] | This project was supported by the Ministry of Defence, Royal Navy. |
| Nye 2016[32] | Not reported |
| O’Connor 2011[33] | Research grant award from the American Medical Society for Sports Medicine. |
| Orr 2020[34] | This study was funded under ATO/Task Area R.MRM.2010.01. |
|  | This study was funded under ATO/Task Area R.MRM.2010.01. |
| Roos 2015[35] | Not reported |
| Schram 2019i[36] | This study was partially funded by a Defence Health Foundation Grant. |
|  | This study was partially funded by a Defence Health Foundation Grant. |
| Sharma 2011[37, 38] | This research is part of Ph.D. study at Teesside Universityfunded by Army Recruiting and Training Division, Ministry ofDefence, UK. |
| Sharma 2015[39, 40] | This work was sponsored by the Army Recruiting and Training Division, UK. |
| Trone 2014[41] | ND |
| Withnall 2006[42] | Defence Post Graduate Medical Deanary in part |
| Wyss 2012[43] | ND |
| Wyss 2014[44] | ND |

**Appendix F. Generalised linear model of study level data, assessing the association between the medical attention injury incidence rate (95% CI), and the duration of the recruit training program (weeks).**

| **Variable** | **β-estimate** | **95% confidence interval** | **p-value** |
| --- | --- | --- | --- |
| Time (weeks) | -0.373 | -0.616 to -0.131 | 0.003 |

**Appendix G. Quality of include studies**

| **Study** | **1. Was the sample frame appropriate to address the target population?** | **2. Were study participants sampled in an appropriate way?** | **3. Was the sample size adequate?** | **4. Were the study subjects and the setting described in detail?** | **5. Was the data analysis conducted with sufficient coverage of the identified sample?** | **6. Were valid methods used for the identification of the condition?** | **7. Was the condition measured in a standard, reliable way for all participants?** | **8. Was there appropriate statistical analysis?** | **9. Was the response rate adequate, and if not, was the low response rate managed appropriately?** | **Overall Judgement** |
| --- | --- | --- | --- | --- | --- | --- | --- | --- | --- | --- |
| Billings 2004[40] | Yes | Yes | Yes | Yes | Yes | Yes | Yes | N/A | Yes | High |
| Blacker 2008[11] | Yes | Yes | Yes | Yes | Yes | Yes | Yes | N/A | Yes | High |
| Booth 2006[3] | No | Yes | No | No | Unclear | Unclear | Unclear | N/A | Unclear | Low |
| Brooks 2019[41] | Yes | Yes | Yes | No | Unclear | Yes | Yes | N/A | Yes | High |
| Brushøj 2008[42] | Yes | Yes | Yes | No | Yes | Yes | Yes | N/A | Yes | High |
| Burley 2020[43] | No | Yes | No | No | Yes | Unclear | Yes | N/A | Yes | High |
| Chassé 2020[16] | Yes | Yes | Yes | No | Yes | Yes | Yes | N/A | Yes | High |
| Cowan 2011[15, 37] | Yes | No | Yes | No | Yes | Yes | Yes | N/A | Yes | High |
| Cowan 2012[44] | Yes | Yes | Yes | No | Yes | Yes | Yes | N/A | Yes | High |
| Dawson 2015[45] | Yes | Yes | No | No | Yes | No | Yes | N/A | Yes | Low |
| Din 2016[46] | No | No | Yes | Yes | Yes | Yes | Yes | N/A | Yes | High |
| Esterman 2005[17] | No | No | No | No | Yes | Unclear | Unclear | N/A | Yes | Low |
| Everard 2018[47] | No | No | No | Yes | Unclear | Yes | Yes | N/A | Unclear | Low |
| Fallowfield 2020[12] | Yes | Yes | Yes | No | Yes | Yes | Yes | N/A | Yes | High |
| Goodall 2013[48] | Yes | Yes | No | No | Yes | Yes | Yes | N/A | Yes | High |
| Hall 2017[13] | Yes | Yes | Yes | No | Yes | Unclear | Unclear | N/A | Yes | Low |
| Hauschild 2018[8] | Yes | Yes | Yes | No | Yes | Yes | Yes | N/A | Yes | High |
| Havenetidis 2011[49] | Unclear | Unclear | No | Yes | Unclear | Yes | Yes | N/A | Unclear | Low |
| Havenetidis 2017[50] | Unclear | Unclear | No | Yes | Yes | Yes | Yes | N/A | Yes | Low |
| Heagerty 2018[18] | Yes | Yes | Yes | No | Unclear | Yes | Yes | N/A | Yes | Low |
| Heller 2020[4] | Yes | Yes | No | No | Yes | Yes | Yes | N/A | Yes | High |
| Hofstetter 2012[51] | Unclear | Yes | No | Yes | Yes | Yes | Yes | N/A | Yes | High |
| Jones 2017[19] | Yes | Yes | Yes | Yes | Yes | Yes | Yes | N/A | Yes | High |
| Kerr 2004[52] | Yes | Yes | No | No | Yes | Yes | Unclear | N/A | Yes | Low |
| Knapik 2006[54] | Yes | Yes | Yes | No | Yes | Yes | Yes | N/A | Yes | High |
| Knapik 2010a[53] | Unclear | Yes | Yes | No | Yes | Yes | Yes | N/A | Yes | High |
| Knapik 2010b[55] | Unclear | Yes | Yes | No | Yes | Yes | Yes | N/A | Yes | High |
| Mohammadi 2013[56] | No | No | No | Yes | Yes | Yes | Yes | N/A | Yes | Low |
| Müller-Schilling 2019[5] | Unclear | No | Yes | No | Unclear | Yes | Unclear | N/A | Unclear | Low |
| Munnoch 2007[57] | Yes | Yes | Yes | No | Yes | Unclear | Unclear | N/A | Yes | Low |
| Nye 2016[22] | Yes | Yes | Yes | No | Yes | Yes | Yes | N/A | Yes | High |
| O’Connor 2011[20] | Unclear | Yes | Yes | No | Yes | Yes | Yes | N/A | Yes | High |
| Orr 2020[14] | Yes | Yes | Yes | No | Yes | Yes | Yes | N/A | Yes | High |
| Roos 2015[6] | Yes | Yes | No | Yes | Yes | Yes | Yes | N/A | Yes | High |
| Schram 2019[9] | Yes | Yes | Yes | No | Unclear | No | Yes | N/A | Yes | Low |
| Sharma 2011[7, 36] | Unclear | Unclear | No | Yes | Unclear | Yes | Yes | N/A | Yes | Low |
| Sharma 2015[10, 35] | Yes | Yes | Yes | Yes | Unclear | Yes | Yes | N/A | Yes | High |
| Trone 2014[58] | Yes | Yes | Yes | Yes | Yes | Yes | Yes | N/A | Yes | High |
| Withnall 2006[59] | Unclear | No | No | Yes | Unclear | Yes | Unclear | N/A | Yes | Low |
| Wyss 2012[61] | Yes | Yes | No | No | Yes | Yes | Yes | N/A | Yes | High |
| Wyss 2014[60] | Unclear | Yes | Yes | No | Yes | Yes | Yes | N/A | Yes | High |

**REFERENCES**

1. Chassé E, Laroche MA, Dufour CA, Guimond R, Lalonde F. Association Between Musculoskeletal Injuries and the Canadian Armed Forces Physical Employment Standard Proxy in Canadian Military Recruits. Military medicine. 2020;185(7-8):e1140-e6.

2. Kerr GM. Injuries sustained by recruits during basic training in Irish Army. Irish medical journal. 2004;97(3):80-1.

3. Esterman A, Pilotto L. Foot shape and its effect on functioning in Royal Australian Air Force recruits. Part 1: Prospective cohort study. Military medicine. 2005;170(7):623-8.

4. Fallowfield JL, Leiper RG, Shaw AM, Whittamore DR, Lanham-New SA, Allsopp AJ, et al. Risk of Injury in Royal Air Force Training: Does Sex Really Matter? Military medicine. 2020;185(1-2):170-7.

5. Billings CE. Epidemiology of injuries and illnesses during the United States Air Force Academy 2002 Basic Cadet Training program: documenting the need for prevention. Military medicine. 2004;169(8):664-70.

6. Blacker SD, Wilkinson DM, Bilzon JL, Rayson MP. Risk factors for training injuries among British Army recruits. Military medicine. 2008;173(3):278-86.

7. Booth CK, Probert B, Forbes-Ewan C, Coad RA. Australian army recruits in training display symptoms of overtraining. Military medicine. 2006;171(11):1059-64.

8. Brooks RD, Grier T, Dada EO, Jones BH. The Combined Effect of Cigarette Smoking and Fitness on Injury Risk in Men and Women. Nicotine & tobacco research : official journal of the Society for Research on Nicotine and Tobacco. 2019;21(12):1621-8.

9. Brushøj C, Larsen K, Albrecht-Beste E, Nielsen MB, Løye F, Hölmich P. Prevention of overuse injuries by a concurrent exercise program in subjects exposed to an increase in training load: a randomized controlled trial of 1020 army recruits. American journal of sports medicine. 2008;36(4):663‐70.

10. Burley SD, Drain JR, Sampson JA, Nindl BC, Groeller H. Effect of a novel low volume, high intensity concurrent training regimen on recruit fitness and resilience. Journal of science and medicine in sport. 2020;23(10):979-84.

11. Bedno SA, Cowan DN, Urban N, Niebuhr DW. Effect of pre-accession physical fitness on training injuries among US Army recruits. Work (Reading, Mass). 2013;44(4):509-15.

12. Cowan DN, Bedno SA, Urban N, Yi B, Niebuhr DW. Musculoskeletal injuries among overweight army trainees: incidence and health care utilization. Occupational medicine (Oxford, England). 2011;61(4):247-52.

13. Cowan DN, Bedno SA, Urban N, Lee DS, Niebuhr DW. Step test performance and risk of stress fractures among female army trainees. American journal of preventive medicine. 2012;42(6):620-4.

14. Dawson GME, Broad R, Orr RM. The impact of a lengthened Australian Army recruit training course on recruit injuries. Journal of Military & Veterans' Health. 2015;23(2):14-9.

15. Din FHM, Rampal S, Muslan MA, Hoe VCW. Association between pain catastrophising and musculoskeletal disorders is modified by past injuries in Malaysian military recruits. Occupational and Environmental Medicine. 2016;73(7):429-34.

16. Everard E, Lyons M, Harrison AJ. Examining the association of injury with the Functional Movement Screen and Landing Error Scoring System in military recruits undergoing 16 weeks of introductory fitness training. Journal of science and medicine in sport. 2018;21(6):569-73.

17. Goodall RL, Pope RP, Coyle JA, Neumayer R. Balance and agility training does not always decrease lower limb injury risks: a cluster-randomised controlled trial. International journal of injury control and safety promotion. 2013;20(3):271-81.

18. Hall LJ. Relationship between 1.5-mile run time, injury risk and training outcome in British Army recruits. J R Army Med Corps. 2017;163(6):376-82.

19. Hauschild VD, Lee T, Barnes S, Forrest L, Hauret K, Jones BH. The Etiology of Injuries in US Army Initial Entry Training. US Army Medical Department journal. 2018(2-18):22-9.

20. Havenetidis K, Kardaris D, Paxinos T. Profiles of musculoskeletal injuries among Greek Army officer cadets during basic combat training. Military medicine. 2011;176(3):297-303.

21. Havenetidis K, Paxinos T, Kardaris D, Bissas A. Prognostic potential of body composition indices in detecting risk of musculoskeletal injury in army officer cadet profiles. The Physician and sportsmedicine. 2017;45(2):114-9.

22. Heagerty R, Sharma J, Cayton J, Goodwin N. Retrospective analysis of four-year injury data from the Infantry Training Centre, Catterick. J R Army Med Corps. 2018;164(1):35-40.

23. Heller R, Stammers H. Running to breaking point? The relationship between 1.5-mile run time and injury risk in female recruits during British Army basic training. Bmj Military Health. 2020;166(E):E3-E7.

24. Hofstetter MC, Mäder U, Wyss T. Effects of a 7-week outdoor circuit training program on Swiss Army recruits. Journal of strength and conditioning research. 2012;26(12):3418-25.

25. Jones BH, Hauret KG, Dye SK, Hauschild VD, Rossi SP, Richardson MD, et al. Impact of physical fitness and body composition on injury risk among active young adults: A study of Army trainees. Journal of science and medicine in sport. 2017;20 Suppl 4:S17-s22.

26. Knapik JJ, Darakjy S, Hauret KG, Canada S, Scott S, Rieger W, et al. Increasing the physical fitness of low-fit recruits before basic combat training: an evaluation of fitness, injuries, and training outcomes. Military medicine. 2006;171(1):45-54.

27. Knapik JJ, Brosch LC, Venuto M, Swedler DI, Bullock SH, Gaines LS, et al. Effect on injuries of assigning shoes based on foot shape in air force basic training. American journal of preventive medicine. 2010;38(1 Suppl):S197-211.

28. Knapik JJ, Trone DW, Swedler DI, Villasenor A, Bullock SH, Schmied E, et al. Injury reduction effectiveness of assigning running shoes based on plantar shape in Marine Corps basic training. The American journal of sports medicine. 2010;38(9):1759-67.

29. Mohammadi F, Azma K, Naseh I, Emadifard R, Etemadi Y. Military exercises, knee and ankle joint position sense, and injury in male conscripts: a pilot study. Journal of athletic training. 2013;48(6):790-6.

30. Müller-Schilling L, Gundlach N, Böckelmann I, Sammito S. Physical fitness as a risk factor for injuries and excessive stress symptoms during basic military training. International archives of occupational and environmental health. 2019;92(6):837-41.

31. Munnoch K, Bridger RS. Smoking and injury in Royal Marines' training. Occupational medicine (Oxford, England). 2007;57(3):214-6.

32. Nye NS, Pawlak MT, Webber BJ, Tchandja JN, Milner MR. Description and Rate of Musculoskeletal Injuries in Air Force Basic Military Trainees, 2012-2014. Journal of athletic training. 2016;51(11):858-65.

33. O'Connor FG, Deuster PA, Davis J, Pappas CG, Knapik JJ. Functional movement screening: predicting injuries in officer candidates. Medicine and science in sports and exercise. 2011;43(12):2224-30.

34. Orr RM, Cohen BS, Allison SC, Bulathsinhala L, Zambraski EJ, Jaffrey M. Models to predict injury, physical fitness failure and attrition in recruit training: a retrospective cohort study. Military Medical Research. 2020;7(1):26.

35. Roos L, Boesch M, Sefidan S, Frey F, Mäder U, Annen H, et al. Adapted marching distances and physical training decrease recruits' injuries and attrition. Military medicine. 2015;180(3):329-36.

36. Schram B, Pope R, Orr R. Injuries in Australian Army full-time and part-time personnel undertaking basic training. BMC musculoskeletal disorders. 2019;20(1):6.

37. Sharma J, Golby J, Greeves J, Spears IR. Biomechanical and lifestyle risk factors for medial tibia stress syndrome in army recruits: a prospective study. Gait & posture. 2011;33(3):361-5.

38. Sharma J, Heagerty R, Dalal S, Banerjee B, Booker T. Risk Factors Associated With Musculoskeletal Injury: A Prospective Study of British Infantry Recruits. Current rheumatology reviews. 2019;15(1):50-8.

39. Sharma J, Dixon J, Dalal S, Heagerty R, Spears I. Musculoskeletal injuries in British Army recruits: a prospective study of incidence in different Infantry Regiments. J R Army Med Corps. 2017;163(6):406-11.

40. Sharma J, Greeves JP, Byers M, Bennett AN, Spears IR. Musculoskeletal injuries in British Army recruits: a prospective study of diagnosis-specific incidence and rehabilitation times. BMC musculoskeletal disorders. 2015;16:106.

41. Trone DW, Cipriani DJ, Raman R, Wingard DL, Shaffer RA, Macera CA. Self-reported smoking and musculoskeletal overuse injury among male and female U.S. Marine Corps recruits. Military medicine. 2014;179(7):735-43.

42. Withnall R, Eastaugh J, Freemantle N. Do shock absorbing insoles in recruits undertaking high levels of physical activity reduce lower limb injury? A randomized controlled trial. Journal of the Royal Society of Medicine. 2006;99(1):32-7.

43. Wyss T, Von Vigier RO, Frey F, Mäder U. The Swiss Army physical fitness test battery predicts risk of overuse injuries among recruits. The Journal of sports medicine and physical fitness. 2012;52(5):513-21.

44. Wyss T, Roos L, Hofstetter MC, Frey F, Mäder U. Impact of training patterns on injury incidences in 12 Swiss Army basic military training schools. Military medicine. 2014;179(1):49-55.
